# The Importance of Clinical Mentorship Through the Lens of Newly‐Graduated Registered Nurses: A Focused Ethnographic Study in a Hospital Setting

**DOI:** 10.1111/jocn.70339

**Published:** 2026-05-13

**Authors:** Pernilla Berndtsson, Malin Berghammer, Lars Walter, Maria Skyvell Nilsson

**Affiliations:** ^1^ Department of Health Sciences University West Trollhättan Sweden; ^2^ The Queen Silvia Children's Hospital Gothenburg Sweden; ^3^ Department of School of Business and IT University West Trollhättan Sweden

**Keywords:** clinical mentors, focused ethnography, hospital settings, newly‐graduated registered nurses, nursing expertise

## Abstract

**Aim:**

To explore newly‐graduated registered nurses' professional needs and how these needs are supported by mentors in clinical practice, at two Swedish hospital care units.

**Background:**

Previous studies show that newly‐graduated registered nurses face challenges on initially starting to provide hospital care, thus needing organisational support. Experienced nurses and mentors are commonly described in the literature as facilitating this support and need to be investigated further; however, where this appears in a hospital setting.

**Design/Method:**

A qualitative study using focused ethnography was conducted between May 2024 and March 2025. Data was generated from 9 different fieldwork sessions (68 h) for mentoring that included newly‐graduated registered nurses and mentors from two different care units at hospitals within one regional healthcare authority in Sweden. Data was collected by means of participant observations, shadowing, ethnographic interviews, and the use of ethnographic analysis.

**Findings:**

One main theme was identified: *I'm in freefall and in need of practical, social and emotional support when navigating my new role,* as well as two subthemes: (1) I am a new nurse and in need of help in comprehending and performing nursing, and (2) I am undergoing a learning process in need of a trust‐based relationship. Each theme includes three subthemes.

**Conclusion:**

Newly‐graduated registered nurses need consistent practical, social, and emotional support in their day‐to‐day work through trust‐based relationships with mentors. This is a relationship crucial for their learning process, and which helps them bridge the gap between understanding and practicing nursing.

**Implications for the Profession:**

Structured mentorship can improve the learning environment of newly‐graduated registered nurses and constitute a sustainable working environment for them. Decision‐makers and managers can use this knowledge to implement mentoring programmes that are of interest when it comes to retaining both new and experienced nurses and providing qualitative and safe care.

**Reporting Method:**

This study conforms to the reporting of the COREQ guidelines and checklist.

**Patient or Public Contributions:**

No patient or public contributions.

## Summary

- Which problem did the study address?
  ○ Newly‐graduated registered nurses face a challenging time during their first year as newcomers to the profession.
  ○ There is a lack of evidence‐based mentor programmes for newly‐graduated registered nurses.
- What were the main findings?
  ○ In their day‐to‐day work, newly graduated‐registered nurses use the support of their clinical mentors to navigate between developing an understanding of and performing nursing practice.
  ○ A trust‐based relationship is essential for both identifying the newly‐graduated registered nurses' individual needs and building a meaningful learning process.
- Where and on whom will the research have an impact?
  ○ Decision‐makers in healthcare organisations when it comes to improving newly‐graduated registered nurses' competence.
  ○ Nursing managers when it comes to developing more targeted mentorship programmes.
- What does this paper contribute to the wider global clinical community?
  ○ This study contributes to the understanding of how a trusting and responsive mentor relationship can provide practical, social and emotional support for newly‐graduated nurses in their clinical practice.

## 1 Introduction

Nurses represent the largest professional group in healthcare and play a vital role in delivering high‐quality patient care (World Health Organization 2020). In 2020, the global workforce in healthcare comprised 65 million, with about one‐third of these being nurses and in 2030, it is estimated that an additional nine million nurses and midwives will be needed (Boniol et al. 2022). This need for nurses highlights the importance of their wellbeing and the urgent need for strategies for retaining them within both clinical practice and the profession. However, a study conducted in three European countries indicates that the support provided to newly‐graduated registered nurses (NGRNs) is insufficient, with findings highlighting barriers within the clinical learning environment, poor working conditions, and negative interpersonal relationships in the workplace (Aleo et al. 2025). In line with this a Swedish study, by Eklund et al. (2025), found that NGRNs' high workload, the burden of being responsible for too many patients, and insufficient learning opportunities constitute major challenges. Further, NGRNs often lack the support of their experienced colleagues and opportunities to ask questions in their day‐to‐day work, something which has been identified as a factor contributing towards nurses leaving their current workplaces (Berthelsen and Hansen 2024). The difficulties faced by NGRNs in adapting to a demanding new working environment can lead to burnout, stress, and perceived job difficulties, which are all associated with the intention to leave the profession (Lyu et al. 2024).

Understanding NGRNs' early professional development can be framed using Benner (1982) model of skill acquisition, which involves five stages: *novice, advanced beginner, competent*, *proficient* and *expert*. Novice nurses are undergoing their nursing education, have acquired generic theoretical knowledge, with advanced beginners having entered clinical practice, learning theory that is context‐free during their education alongside practice in the workplace. They need to manage real situations and learn what is relevant to their skills in practice (i.e., situational learning), while also relying on rules and guidelines, and focusing on remembering procedures and rules. Competent nurses are skilled in understanding the interrelationships between actions and their consequences, enabling them to plan their care while taking into account their patients' long‐term goals. Further, proficient nurses can also interpret situations as integrated wholes: Experts no longer depend on explicit rules, they draw on extensive prior experience and intuition when identifying problems and implementing appropriate, holistic nursing interventions (Benner 1982). Novices and advanced beginners (i.e., NGRNs) need to integrate with mentors who are experienced nurses on a competent level at the very least (Benner 1984).

Research highlights mentorship as a key strategy for improving the retention of NGRNs (De Vries et al. 2023; Södergård et al. 2025), with the relationship between mentor and mentee being described as vital for retention (Vázquez‐Calatayud and Eseverri‐Azcoiti 2023). According to NGRNs, when personal mentors provide support and opportunities for clinical skill development, thus rendering the initial transition into professional practice both educational and professionally rewarding (Tast et al. 2024). Mentors are reported to play a key role in enhancing NGRNs' confidence in patient care while promoting a welcoming and inclusive workplace (Hallaran et al. 2023). In addition, Alharbi et al. (2023) have described how dedicated mentors supporting NGRNs in developing the necessary knowledge and skills have positively impacted their adaptation to the hospital setting. Further, a scoping meta‐review has shown mentorship programmes to be beneficial for mentees' learning, growth, development, and increased confidence and competence, while the huge variation in duration and structure has made it difficult to evaluate these mentoring programmes (Giltenane et al. 2026).

In summary, while the literature highlights the multiple benefits of mentoring, studies indicate that the quality and structure of the mentoring support provided to NGRNs are influenced by healthcare leadership and organisational prerequisites within the clinical setting (Berndtsson et al. 2024; Kallerhult Hermansson et al. 2026). This reveals the need for exploring mentorship, as a cultural phenomenon, more closely in everyday clinical practice. In investigating this cultural phenomenon, an ethnographic approach is considered appropriate, as it allows researchers to describe and understand why people behave as they do in natural settings (Roper and Shapira 2000), thus generating knowledge that can be applied in order to develop more targeted mentorship programmes.

## 2 Aim

The aim of this study was to explore NGRNs' professional needs and how these needs are supported by mentors in clinical practice at two Swedish hospital care units.

## 3 Method

### 3.1 Design, Setting, Sample and Data Collection

Focused ethnography design is appropriate when studying a specific cultural perspective on subgroups of people in a specific context (Higginbottom et al. 2013). The focus of this study was mentorship within a hospital culture in Sweden. Focused ethnography, including focused observations spending a relatively short time in the field, was also considered suitable as the researcher was familiar with and had extensive knowledge of the field (Andreassen et al. 2020). Given the limited amount of time in the field, careful and deliberate planning was implemented before, during, and after the fieldwork in accordance with Andreassen et al. (2020), to ensure that data collection was focused, systematic, and rigorous.

The specific setting for this study was two Swedish hospital care units within a regional health authority, offering NGRNs the opportunity to participate in a 1‐year hospital introductory programme that included training opportunities, regular group meetings for reflection, seminar training and an introduction that was specific to the care unit. In addition, some care units had also implemented mentorship as a complement to their introduction programmes, during which experienced or senior nursing advisors offered support, in the present study defined as ‘clinical mentors’.

The study focused on structured mentorship that had been established and sanctioned by care unit managers. Two care units were selected at two different hospitals; one, a university hospital with a paediatric setting, and the other, a county hospital specialising in surgery. Senior managers and care unit managers were informed via email, and their approval was obtained. The contact details of five clinical mentors were then collected. The clinical mentors were informed about the study (by P.B.), both via email and verbally, and consent was obtained. The NGRNs at the two care units were informed about the study by researcher P.B. and asked for their consent, and all the participants chose to participate. In this study, the term NGRN refers to registered nurses who have recently completed their undergraduate education, holding a 3‐year bachelor's degree. These NGRNs had graduated up to 12 months prior to data collection, and the clinical mentors were expected to have prior mentoring experience, although no specific duration was required (see Table 1).

**TABLE 1:** Newly‐graduated registered nurses and mentors observed during fieldwork.

	NGRNs (*n* = 10)	Mentors (*n* = 5)
Female/Male	10/0	4/1
Age (years)	23–50	34–49
Experience as nurses (years)	< 0.67*	12–27
Experience as mentors (years)	0	1–8

* The NGRNs had between 2 weeks and 8 months of experience as nurses.

All the participating clinical mentors had extensive experience as nurses, were well known within their respective care units' nursing speciality and held a formal role as mentors with time allocated to supporting NGRNs. The clinical mentors rotated, being rostered to work as both mentors and nurses responsible for patients of their own. Mentoring was commonly offered during the daytime, in the form of support during day‐to‐day work, mentoring conversations, or group reflection sessions. The mentors independently decided how to provide support to the NGRNs in their day‐to‐day work, tailoring their approach to the individual needs of each nurse, the specific demands of the unit, and the challenges that arose during everyday clinical practice. Overall, mentors' assignments were similar across the care units. However, at the surgical unit, work was characterised by high patient throughput, requiring mentors to constantly guide and motivate their NGRNs to facilitate patient discharges. In contrast, this focus was less evident at the paediatric unit, where patient stays were generally longer.

In order to explore the NGRNs' professional needs and how these needs were supported by mentors during clinical practice, the clinical mentors and NGRNs were seen as key participants in accordance with Higginbottom et al. (2013), possessing in‐depth knowledge and experience of the topic in focus. Data collection was carried out between May 2024 and March 2025. A total of nine fieldwork sessions (in total 68 h) were completed by the first author (P.B.) and given a numeric code (Fieldwork sessions 1, 2, 3, etc.). Each fieldwork session lasted between 4 and 9.5 h (see Table 2) and was carried out mostly during the daytime, but also during major public holidays. During data collection, the locations of the observations were not predetermined as it was necessary to adapt both to the mentoring situations that arose and to the participants involved. Data was collected using several methods: *participant observations*, *shadowing* and *ethnographic interviews*. This diversity of methods helped the researchers to gather the rich data specifically needed for focused ethnography, where data collection is intensive (Andreassen et al. 2020). The observations focused on mentorship in practice, as well as the interaction between mentors and NGRNs.

**TABLE 2:** Overview of fieldwork sessions and interviews.

Care unit and fieldwork sessions	Participants (***n***)	Time spent on the field session (h)	Number of interviews with NGRNs
Paediatric care unit			
1	Mentor (1); NGRNs (3)	7	
2	Mentor (1); NGRNs (3)	4	
3	Mentors (3); NGRNs (4); assistant nurse (1)	5.5	
4	Mentor (1); NGRNs (3)	8.5	2
5	Mentor (1); NGRNs (3)	9.5	2
Surgical care unit			
6	Mentors (2); NGRNs (1)	9.5	1
7	Mentor (1); NGRNs (1)	8.5	1
8	Mentor (1); NGRNs (1)	8.5	1
9	Mentor (1)	7	
Total: 9 occasions	Total: 5 mentors; 10 NGRNs *	Total: 68 h	Total: 7

* Informal conversations were conducted with NGRNs and mentors.

A shadowing approach was employed (McDonald 2005), at the beginning of the fieldwork sessions whereby P.B. followed the clinical mentors to gain some insight into their role. This approach subsequently transitioned into participant observations inspired by Atkinson and Pugsley (2005), applied during interactions and collaborations between the clinical mentors and NGRNs. As an observer, P.B. was dressed in a nurse's uniform and able to assist when needed in specific situations. Fieldnotes were taken continuously during the fieldwork sessions in order to describe the specific situations by means of describing the place, time, people involved, atmosphere and tempo, as well as the situations occurring and how the clinical mentors and NGRNs interacted with each other verbally. This also included what was said, by whom, to whom and in what way, as well as how they acted in a specific situation and what their expressions of emotion were. All the fieldnotes were written as soon after each fieldwork session as possible.

During the fieldwork sessions, reflexive interviews were conducted, as inspired by Hammersley and Atkinson (1989). These interviews were shaped as informal conversations and were guided by questions arising during the observations initiated by the researcher close in time to an activity, in order to clarify an observed situation or a statement by an NGRN and/or a mentor. An individual interview was conducted with an NGRN in rooms not subject to disturbance. This interview started with informal questions about what had been observed during the day, for instance; *Now, when you were having mentor conversations, would you tell me what was of key importance during these conversations?* In the next step, questions were more structured in terms of focusing on their experiences of mentoring at the care unit; *Would you tell me about a previous situation where you had the support of the mentors? Would you tell me about a previous situation when you had lacked, and wished you had received, your mentor's help?* These questions were asked to ensure that it was the commonly‐occurring situations in their day‐to‐day work that the NGRNs needed support with that is, not unusual situations (Atkinson and Pugsley 2005). Seven interviews were audio recorded, between 16 and 31 min (mean = 27 min), together with the fieldnotes (including a total of 110 pages and 41,100 words), transcribed verbatim into Swedish, and serving as the data for our analysis.

During one planned mentoring session involving scenario training, it was noted, after the session, that one participant had been a nurse who was a newcomer to the unit but who had prior nursing experience (of about 3 years). However, nothing remarkable was observed in connection with this person's needs or the support provided by the mentor.

### 3.2 Ethical Considerations

This study was reviewed by the Swedish Ethical Review Authority (no. 2022‐00111‐01) and has followed good research practice, in line with the Declaration of Helsinki (World Medical Association 2013). When signing the consent form, all the participants were informed about the voluntary nature of the study and that they would be able to terminate their participation at any time without providing a reason. Patients and relatives were not included since the focus of the study was the interactions between the NGRNs and their clinical mentors. Colleagues taking part in the fieldwork mentoring sessions, for example, assistant nurses and doctors, were not in focus in the context of this study, and thus verbal consent was obtained from them (one assistant nurse attending the group mentoring sessions had given her informed consent). Participant confidentiality was taken into consideration throughout all the stages of the research process. To safeguard the participants' anonymity, pseudonyms were used for all the participants during data collection, transcription and analysis. In order to publish the findings of the study, the NGRNs were given fictitious names, and the clinical mentors were named as mentors.

### 3.3 Data Analysis

**PHOTO 1:**
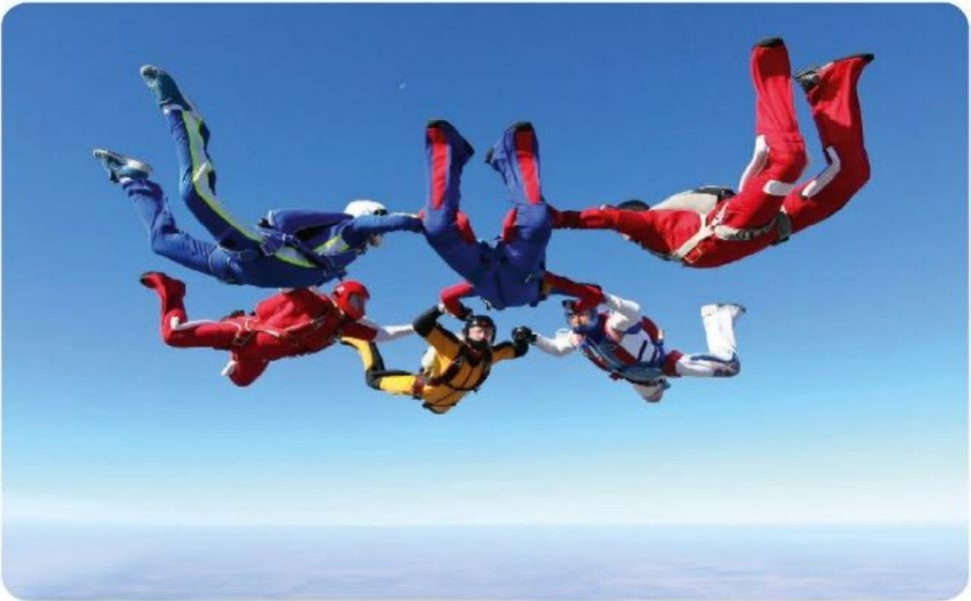
The photo illustrates NGRNs' feeling of being in ‘free fall’, and their need for support to be close at hand. Photo: Resultatbolaget. [Colour figure can be viewed at wileyonlinelibrary.com]

**PHOTO 2:**
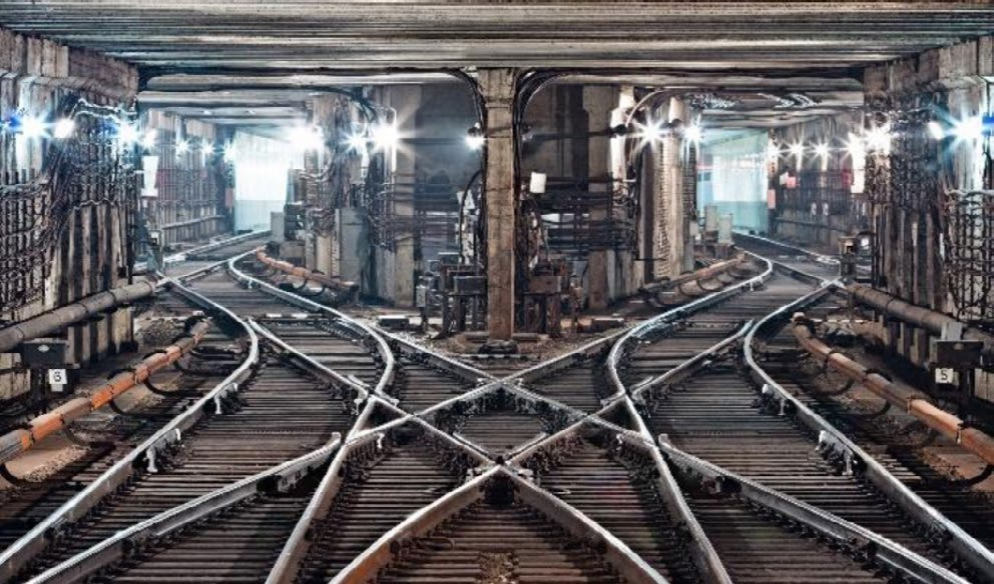
The photo illustrates NGRNs' perceptions of being in unfamiliar situations, of not understanding what appropriate actions would be. Photo: Resultatbolaget. [Colour figure can be viewed at wileyonlinelibrary.com]

**PHOTO 3:**
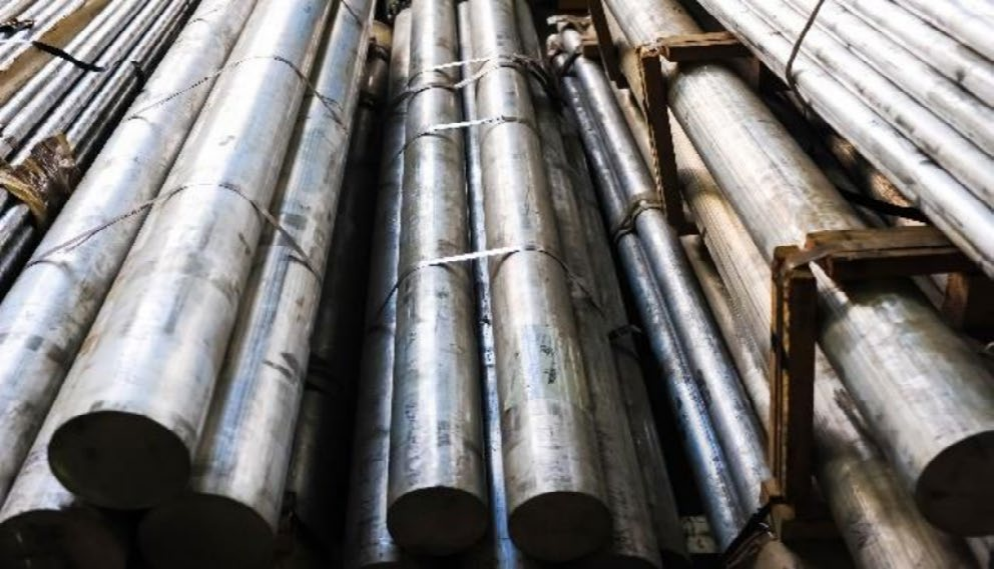
The photo illustrates NGRNs' feeling of being overwhelmed by the choices they have to make. Photo: Resultatbolaget. [Colour figure can be viewed at wileyonlinelibrary.com]

**PHOTO 4:**
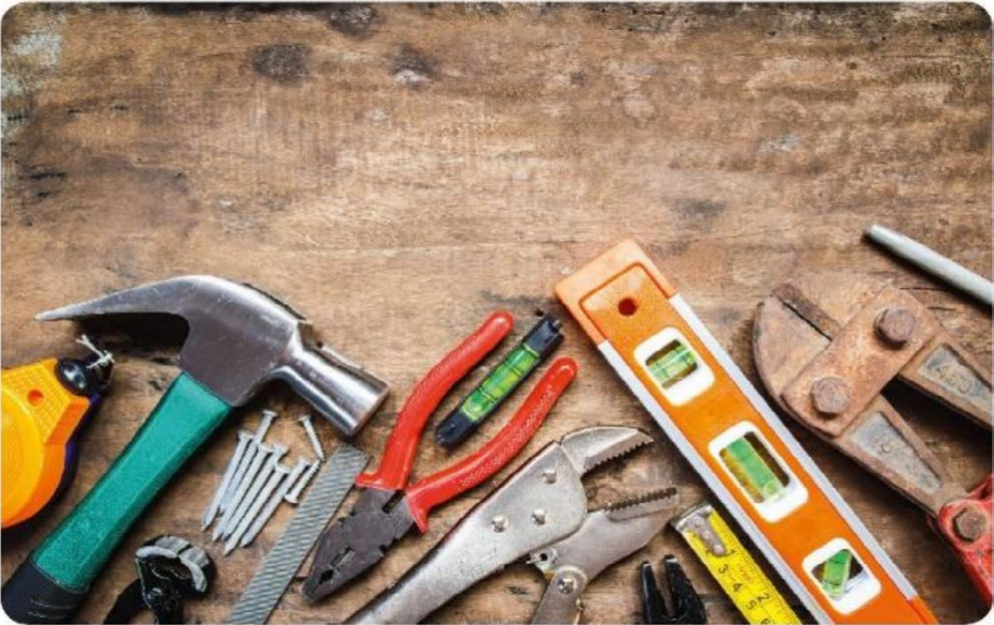
The photo illustrates NGRNs' need for learning strategies and opportunities for further development. Photo: Resultatbolaget. [Colour figure can be viewed at wileyonlinelibrary.com]

**PHOTO 5:**
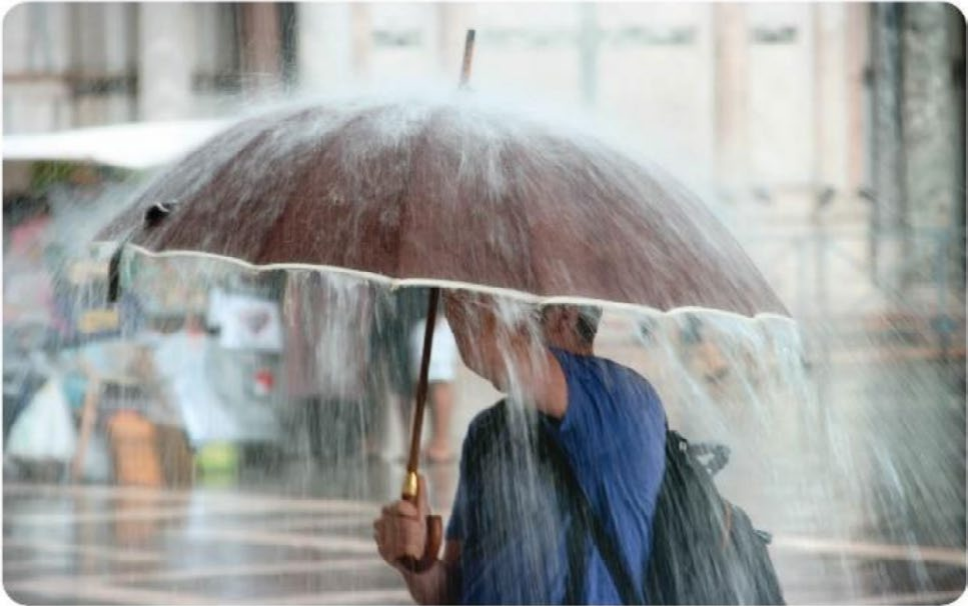
The photo illustrates NGRNs' needs and sensibilities related to receiving feedback regarding their performance. Photo: Resultatbolaget. [Colour figure can be viewed at wileyonlinelibrary.com]

**PHOTO 6:**
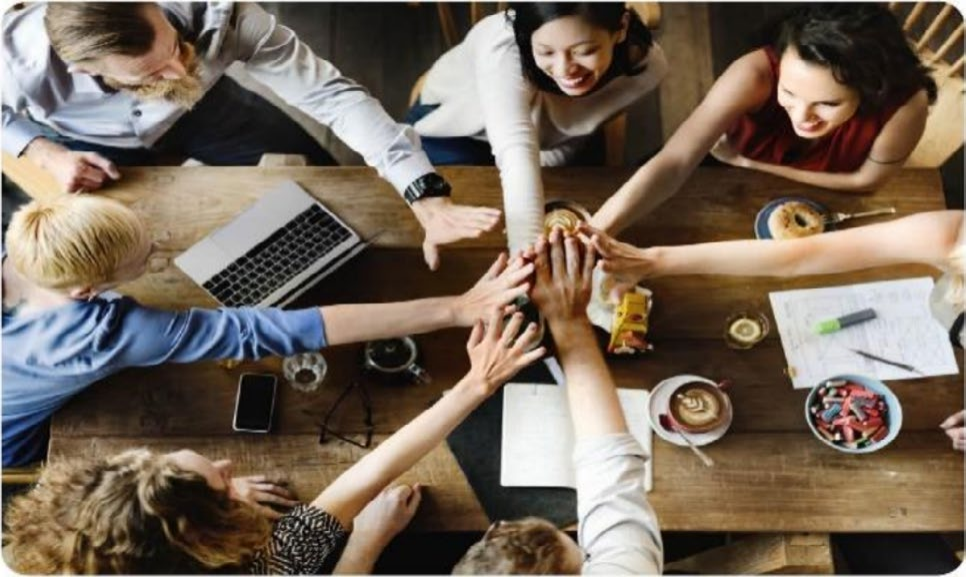
The photo illustrates NGRNs' need for recognition and a sense of belonging at the care unit. Photo: Resultatbolaget. [Colour figure can be viewed at wileyonlinelibrary.com]

The analysis was inspired by Roper and Shapira (2000) description of ethnographic analysis. The focus during analysis was on the NGRNs' professional needs and how such needs were supported by their mentors. First, all the texts were read by P.B. and coded with descriptive labels, closely aligned with the raw data. During one mentoring session, photos from a collage were employed as a reflective pedagogical tool to capture the NGRNs' feelings on that particular day. The photograph most commonly chosen by the NGRNs was incorporated into the study's dataset. Consequently, the photographs were also coded as representations of the NGRNs' emotional experiences during clinical practice, as such being considered to indirectly reflect their underlying needs (Photos 1, 2, 3, 4, 5, 6). The analysis proceeded by clustering codes with similar patterns into categories, for example, *difficulties in delegating tasks to colleagues*. This analysis step was carried out in dialogue between P.B., M.S.N. and L.W. Based on the categories, the analysis was refined and interpreted further and sub‐themes were identified, for example, *Showing me how to prioritize and structure my work*. This analysis was guided by the empirical data. By means of an ongoing dialogue within the whole research group, we were able to identify themes for explaining the NGRNs' professional needs and how the mentors engaged with these needs and thus, with further interpretation, an overarching theme was developed. Notetaking on personal reflections was conducted continuously throughout all the phases by P.B., which then were discussed within the research group and complemented by memos, in line with Roper and Shapira (2000), to capture impressions and emerging questions and also to identify preliminary interpretations requiring further exploration, for example, What are these expressions for, and are they visible in other situations? This analysis process was not linear but iterative and, during analysis, it involved movement back and forth between data, interpretations and reflections.

### 3.4 Rigour and Reflexivity

In ethnography research, both the researcher and the research act per se form part of the social world under investigation, requiring careful attention to the researcher's reflexivity in terms of emic (i.e., the perspective of the subject) and etic (i.e., the perspective of the observer/researcher) (Hammersley and Atkinson 1989). The first author (P.B.) was the data‐producing researcher. She is a PhD student and a registered nurse with experience of being both a nurse and a mentor, and familiar with the culture requested in focused ethnography (Andreassen et al. 2020). The researcher's cultural familiarity was acknowledged throughout the data generation and analysis process, even though data collection was conducted outside P.B.'s previous workplace. Cultural familiarity can enhance in depth interpretation and broaden perspectives: However, the risk of overinterpretation was carefully addressed (Alvesson and Sandberg 2021). To manage preconceptions, P.B. maintained some social distance during fieldwork (Hammersley and Atkinson 1989), and initiated analysis by reviewing all the data promptly after each session. P.B. continuously monitored her own assumptions, ensuring that the study remained focused on the participants' perspectives of the mentorship phenomenon rather than on her own interpretations, despite her familiarity with the setting and the knowledge she shared with the participants. To further challenge her preconceptions and ‘make the familiar strange’, reflexive interviews were conducted that enabled the participants to explain and describe their experiences from their own viewpoints (Andreassen et al. 2020). Fieldnotes were kept throughout data collection and analysis and were systematically discussed within the research team to critically examine the emerging interpretations and ensure reflexivity. The research team included senior PhD researchers; that is, two female registered nurses (M.B. and M.S.N.), who were partly familiar with the culture and who were experienced as clinical supervisors, and L.W., a male senior researcher with both a business administration background and experience of ethnographic research in healthcare. The research team's diverse composition and regular discussions actively introduced multiple perspectives based on varied field experience, allowing the empirical data to guide analysis while consciously addressing pre‐understandings. To ensure rigour, we included interview quotes, photos, and excerpts from fieldnotes to support our findings, following the Consolidated Criteria for Reporting Qualitative Research (COREQ) checklist (Tong et al. 2007).

## 4 Findings

**FIGURE 1:**
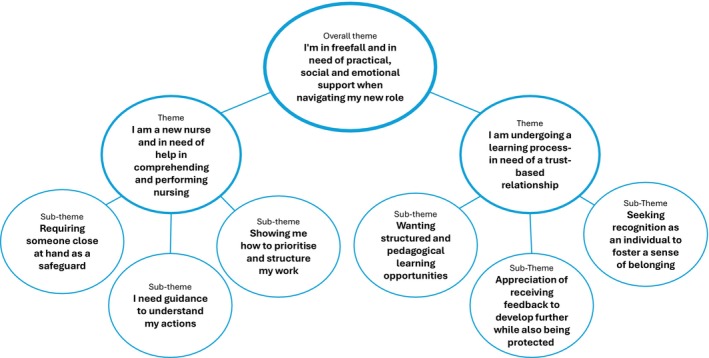
Overview of the findings illustrating the NGRNs' professional needs and how mentors support them in their clinical practice, including an overall theme, two themes and six sub‐themes. [Colour figure can be viewed at wileyonlinelibrary.com]

The findings describe the NGRNs' professional needs and how mentors support them in their clinical practice, summarised in one overall theme: *I'm in freefall and in need of practical, social and emotional support when navigating my new role*. This overall theme captures NGRNs as newcomers to the care unit, lacking situational knowledge while needing to assume the responsibility for their own patients. The NGRNs sought interactions with their clinical mentors who had unit expertise, offered support and teaching, and built trusting relationships that facilitated their learning process of developing as nurses. The overall theme includes two themes that contain a total of six sub‐themes, as described in Figure 1 below.

### 4.1 I Am a New Nurse and in Need of Help in Comprehending and Performing Nursing

This theme illustrates how the NGRNs experience their situation, when expecting to perform nursing, as both exciting and intimidating. They expressed the need to receive continuous practical hands‐on support from their experienced clinical mentors in order to safely comprehend and perform their nursing care. This theme is described using three distinct needs, each addressed by mentors in different ways.

#### 4.1.1 Requiring Someone Close at Hand as a Safeguard

This sub‐theme illustrates the NGRNs' need for the support of their clinical mentors or colleagues to be close at hand while also being expected to be independently responsible for their patient care. The insecurity associated with making immediate situational decisions required having someone to rely on, whereby safeguarding was achieved through the ability to ask questions and receive confirmation of one's own actions. They needed clinical mentors to be present or close at hand as a prerequisite for ensuring patient safety. This need is also illustrated by the photo below (Photo 1), depicting a situation perceived as risky but which includes people to rely on for support.

The NGRNs needed to ask questions to confirm their knowledge before making decisions in real‐life situations. This expectation reflected an approach they had taken with them from their nursing education, where someone was always close at hand to provide support. The NGRNs could just stop working and look for their clinical mentors: for example, during one morning observation, an NGRN only had a short timeframe during which to administer medications before the ward round was to begin:

Johanna is distributing the morning medications from the medication trolley and suddenly looks concerned. She stops reading from the computer, turns around to her mentor and says, ‘I need help here’. She explains that she is unsure about which medications to give as the patient is fasting due to awaiting examinations that day and she then shows the medication list, asking; ‘Which of the morning medications would you have chosen to give to the patient?’ Shortly after, Johanna looks to her mentors again and starts asking questions about another patient's medication, wondering if she should give blood pressure medicine to a patient when that medicine had been put on hold for several days due to low blood pressure (Fieldnote, 8).

The NGRNs were insecure and this was observed, for example, when Lisa began her morning duties accompanied by her mentor, verbalising her focus step‐by‐step as she read the journal and described what she was looking for: ‘Now I'm checking the blood test results’ and ‘Now I see what medication the patient is on’, ‘Now I'm planning to administer that medication’ and so on (Fieldnote, 7). Another NGRN explicitly highlighted the importance of having a clinical mentor available who had the time to listen and validate your thoughts: ‘…thinking out loud with someone who can hear your thoughts and who has the time, that's what mentors have’ (Interview, Sara).

However, the NGRNs felt that the non‐availability of a clinical mentor made it difficult to make decisions that ensure patient safety. Clinical mentors were often available during the daytime, but mostly absent during evening shifts and at weekends, which meant that the NGRNs then had to rely on colleagues. However, a high workload often made colleagues unavailable for answering the NGRNs' questions, creating barriers to obtaining well‐founded answers and reasoning. One NGRN described facing a challenging situation while caring for a patient who had been transferred from the ICU, with multiple medications to administer—including a continuous infusion—with only one of the two tubes working in the central venous catheter: ‘I had to give two medications at the same time, and I didn't know what to do and when I asked a colleague, the answer was too quick, which didn't feel good’ (Interview, Eva).

In summary, the NGRNs wanted a clinical mentor to be available during each shift to provide carefully reasoned answers. Over time, the need for this support tended to decrease as the NGRNs gained in experience and confidence. This type of mentoring is resource‐intensive, requiring close and continuous collaboration between mentors and NGRNs.

#### 4.1.2 I Need Guidance to Understand My Actions

This sub‐theme illustrates the NGRNs' needs as regards their limited knowledge of procedures and clinical judgement, highlighting the need for guidance regarding procedures and reflection on the purposes of their actions. This situation is illustrated by the photo below (Photo 2), selected by the NGRNs as describing their perceptions of their day‐to‐day work.

The NGRNs sought guidance regarding new procedures, and it was observed that the level of guidance needed differed between them. Some NGRNs were keener to rely on their own problem‐solving process, initiating their work independently using the available guidelines. They tested whether or not their problem‐solving strategy was sufficient to perform their duties safely and correctly, seeking guidance from their clinical mentors only when necessary. This is illustrated in the fieldnote below:

Lisa was planning to take blood samples from a percutaneous venous catheter for the first time. She reviewed the nursing instructions on the computer, prepared the materials on the trolley, and repeatedly checked to ensure she had everything she needed. Before entering the patient's room, she double‐checked the materials. Shortly after opening the door, she waved to get her mentor's attention, asking for assistance. The mentor instantly identified some missing items and provided guidance on the need to flush the percutaneous venous catheter before completing the procedure (Fieldnote, 7).

Other NGRNs preferred instead to seek guidance from clinical mentors before acting, feeling unsafe about testing their skills independently, in order to avoid mistakes that could compromise patient safety. During one observation, an NGRN wanted guidance from a clinical mentor with expertise in the clinical situation:

Karin informs her mentor that she's planning to take a blood sample from a central venous catheter that had been difficult before. She is waiting and she wants guidance, with mentors present during the procedure. Before entering the patient's room, the mentors told her that the central venous catheter could, for example, be ‘kinked’ or in need of flushing a little bit faster, also describing what to be observant about (Fieldnote, 4).

Karin later feels that she appreciated her mentor's expertise and guidance:

Get tips on, you know, easier tips, yes ok the mentors usually flush quickly and then sort of aspirate quite quickly, ok then I can try it, it's worked for the mentors, and getting it from the mentors is great, someone who's a bit more experienced (Interview, Karin).

Sometimes, the mentor provided guidance through dialogue in order to improve clinical judgement, to recognise the patient's symptoms, and to understand the purpose of the NGRN's actions, an approach which was appreciated by the new nurses (see fieldnote below).

Lisa is leaving a patient's room, looking stressed and walking with quick and determined steps towards her mentor while saying: ‘The patient is having difficulty breathing’ and then looking at her mentor for a response. The mentor waited before answering her and then Lisa continued by saying: ‘I focused on supplying oxygen’. The mentor then asked calmly: ‘What diagnosis did the patient have again?’ and then ‘What should we think about?’ Lisa started thinking and shortly afterwards the insight occurred to her that it was the pain which was the cause, which the mentor confirmed. Her thinking was right and the mentors was smiling in a friendly way (Fieldnote, 7).

Lisa described later how she had learnt from the situation:

If I hadn't had a mentor, I might not have given enough pain relief to the patient, I might have thought: ‘No, this is OK, it's enough’. However, experienced nurses know that it's a painful condition and that we need to give a bit more pain relief… In dialogue with my mentor, I was able to understand the patient's condition (Interview, Lisa).

In summary, NGRNs need to be guided in order to comprehend and perform their nursing by means of knowledge exchange with nurses with expertise, in reflective dialogue, combining their theoretical knowledge in practice. Yet, NGRNs are individuals who adopt diverse strategies in response to a knowledge deficit: Some are more in need of guidance and support than others, suggesting that clinical mentors need to adapt their mentoring to the individual NGRN and the specific situation.

#### 4.1.3 Showing Me How to Prioritise and Structure My Work

This sub‐theme illustrates how NGRNs expressed the difficulties of structuring their day, organising information, making decisions and prioritising tasks, affecting their ability to lead patient care that is, being overwhelmed by the full range of decisions they are required to make. In doing so, they needed the support of their clinical mentors as regards how to prioritise and organise their work. The NGRNs sometimes appeared overwhelmed, especially when having high workloads, experiencing ‘tunnel vision’, a feeling illustrated by the photo below (Photo 3).

The NGRNs sometimes made decisions that disrupted the flow of care due to difficulties fully grasping the situation. This triggered a process of stress which left them overwhelmed when combined with the challenges of delegating to colleagues and leading care. The clinical mentors identified the NGRNs' feelings and gave advice to those undergoing this stressful process. This was appreciated, as described below:

Karin is working a day shift and there's a high workload at the care unit. During the morning hours, she falls behind in her planning. Before lunchtime, Karin is almost running around the corridor. When her mentors ask how she's doing, she answers: ‘I feel so muddled now’ and she is bright red in her face and neck. Her darting eyes repeatedly read through her handwritten notes, her ‘to‐do lists for the day’, and it's uncertain whether or not she's reading anything, as she appears perplexed. Her mentors say she needs to find the assistant nurse to jointly discuss the plan for the day, and offer to join in their dialogue (Fieldnote, 4).

Karin says afterwards that she appreciated the support from the mentor:

It's very gratifying just being able to get help like structuring things or prioritizing things a bit too, yes it's difficult to prioritize when you're new and it's very good to be able to get help with that. I don't always know what I can get help with (Interview, Karin).

The support of clinical mentors in structuring and prioritising day‐to‐day duties was expressed as vital. The NGRNs valued the guidance provided before situations became overwhelming, as noted in: ‘Mentors ask before things get too stressful and you don't know what to do, so you don't feel like things are too much’ (Interview, Anna). The NGRNs also needed support in order to understand the work structure of their teams, including which duties they could delegate and to whom. Observations also revealed how clinical mentors guided their NGRNs in delegating tasks—for example, dealing with phones, alarms, medications or procedures—to assistant nurses, resource staff, so that the NGRNs could focus on priority duties and thus better structure their day.

In summary, the NGRNs' needs, as regards learning to prioritise and lead their nursing in order to perform work that involves delegating, form an integral part of nursing practice and something to manage rather than to perceive as a failure. However, prioritisation not only requires an awareness of which duties are more important than others; it also includes an understanding of the principles underlying nursing actions in a specific healthcare context.

### 4.2 I Am Undergoing a Learning Process—In Need of a Trust‐Based Relationship

This theme illustrates the NGRNs' need for trust‐based relationships where they can process their feelings of being new in the nursing profession that is, social and emotional support. They also requested pedagogical support, needing confirmation from their clinical mentors during their learning process, something which helps them develop.

#### 4.2.1 Wanting Structured and Pedagogical Learning Opportunities

This sub‐theme highlights the NGRNs' ongoing learning of the prerequisite knowledge of procedures and medical equipment, and as regards handling emergencies. They highlighted the need for diverse pedagogical tools and structured learning opportunities, as illustrated by the photo below (Photo 4).

The NGRNs clearly valued pedagogical learning opportunities, particularly the chance to practice in a safe environment before meeting patients, as expressed in: ‘It feels good to be able to prepare and practice before a real‐world situation arises, and to have the opportunity to go through it first and test it’ (Interview, Anna). The NGRNs appreciated their planned learning activities, as observed when mentors prepared scenario‐based training, allowing them to practice common patient cases and learn from that experience:

After each scenario section, the NGRNs gained the opportunity to reflect on the situation by means of mentors asking questions like: ‘What happened?’ ‘What could we improve?’ ‘What have we learnt from the situation?’ The participants seemed active during the discussion and talked about what they could improve, for instance that they needed to say out loud what they were doing step by step to improve their communication skills and prevent mistakes (Fieldnote, 3).

The NGRNs expressed the need for their clinical mentors to have a respectful attitude towards them as beginners, as well as the pedagogical skills to provide guidance—either using friendly gestures when correcting something in the patient's room or, if necessary, in spoken form for patient safety. The NGRNs expressed the need for mentors who were skilled and willing to answer questions, to provide support and share knowledge. They appreciated it when clinical mentors created learning opportunities as situations arose, as exemplified thus in one interview:

Linda described when a patient became lethargic and tired during an emergency after she had administered medication; she was confused by the situation and wondered what had happened. Is this something we expect from the situation? She had a mentor present who took the opportunity to initiate a learning activity, to say what she was doing out loud so I was able to learn at the same time, the mentor had that mindset (Interview, Linda).

The NGRNs preferred to practice and learn practical skills at their care units, where familiar equipment provided a sense of security: ‘I think it makes a huge difference to be where you're supposed to be… I feel more confident because then I know where my stuff is and what I'm going to do, yes, there's some security in that’ (Interview, Sara). However, the structured learning activities offered to the NGRNs were dependent on time and place, and risked being deprioritised during high workloads, or when there was no space to practice at the care unit.

In summary, the NGRNs appreciated the focused learning activities initiated by the clinical mentors, which could have been difficult for the NGRNs to learn by themselves. This need among the NGRNs emphasises the fact that clinical mentors require expertise and pedagogical skills to plan and identify learning activities, ensuring that the time and space required for learning are prioritised.

#### 4.2.2 Appreciation of Receiving Feedback to Develop Further While Also Being Protected

This sub‐theme illustrates the NGRNs' uncertainty in their new roles, as well as their concerns about having sufficient nursing knowledge, with this occupying their thoughts. They emphasised appreciating feedback in a trustful way in the performance of their work in order to develop further, while also needing to be protected as they were undergoing a learning process while starting up in their profession, as illustrated in the photo below (Photo 5).

The NGRNs clearly wanted feedback in order to develop as nurses and they were receptive to continuous feedback on their work performance, which was usually given at the end of a shift after their clinical mentors had been following them during their day. The NGRNs showed expectations that were mixed with some nervousness at the beginning of these conversations, also wanting to be protected as new nurses and expressing feelings of being nervous about receiving feedback. The NGRNs appreciated it when their clinical mentors reflected on what they had done well, as well as what needed further development, using a trust‐based and gentle approach, which the NGRNs welcomed as a valuable opportunity to receive feedback on their individual competence, thus:

Johanna had a conversation with her mentors who had been observing her during her day and she received her first feedback from her mentor who used a gentle approach regarding her good performance, in addition to some critical feedback about her shortcomings and what needed to be further developed (Fieldnote, 8).

Afterwards, Johanna said: ‘*It's a privilege that the mentor can see me in action and help me think about what I need to develop*’ (Interview, Johanna).

The NGRNs valued feedback from their experienced mentors who had been observing them during their day due to the trust‐based relationship and the mentors' expertise making them feel confident. The NGRNs relied on the mentors' perspectives as regards understanding their learning progress, as they struggled to assess their own performance. This is illustrated below:

If you yourself are undergoing development, you may not see it until you look back on it over a long period of time, so maybe in a year or so, I'll see it but I have really made progress, but she [the mentor] can see it right now from one week to the next… (Interview, Sara).

In summary, the NGRNs are undergoing an intensive learning process, often feeling insecure and needing close support from their clinical mentors. Constructive feedback from their clinical mentors, who have closely been observing them, helps them recognise their progress and build confidence in their professional development.

#### 4.2.3 Seeking Recognition as an Individual to Foster a Sense of Belonging

This sub‐theme illustrates the NGRNs' need for social support and being recognised as individuals, helping to acknowledge them and foster a sense of belonging. They needed to process their feelings in a trust‐based forum through dialogue with their clinical mentors, either individually or in groups, in order to develop as nurses. This need is illustrated in the photo below (Photo 6).

The NGRNs needed a sense of belonging, possibly fostered through group sessions with other NGRNs and a mentor, for example, enabling discussion of and reflection on their learning process. ‘I think that I'm not alone in how I feel as a newcomer, which is common, and you can get tips and ideas on how others do things, so it's very rewarding’ (Interview, Linda).

The NGRNs emphasised the importance of opportunities to have individual conversations with their clinical mentors to discuss their feelings about being newcomers to the profession. During these conversations, they were able to identify and clarify their development process. ‘They keep an eye on you, and I feel that they're interested in checking on how you're feeling, how far you've come in your development, and what you need extra support with’ (Interview, Sara). The NGRNs wanted their mentors to recognise them as individuals and to engage in trust‐based dialogues about their developmental process. They considered it important to be recognised and seen as individuals, with a holistic perspective aligned with the knowledge they had gained during their nursing education, and this was expressed as follows:

It's all so slimmed down and there's so much all the time, and a little extra time for both the new nurse and the person, the human being, that's the new nurse… Without the human being, there wouldn't be a nurse (Interview, Johanna).

The NGRNs' wish to be seen as individuals was also important in the wake of specific difficult incidents, when their clinical mentors had captured their emotions. The following is one example of an NGRN's need for individual support during an observation:

Eva started work during the morning shift, seeing her mentor from a distance in the corridor and then brightening up and going straight to the mentor. This mentor asked how Eva was feeling since she'd heard that she (Eva) had a challenging time of it yesterday with a situation involving a patient and some misunderstandings regarding medicines, with relatives getting upset. Eva was nodding and looking down. The mentor reminded Eva about their previously arranged mentoring conversation, planned for that afternoon, while Eva nodded, smiled slightly, and said that she needed this conversation with her mentors today (Fieldnote, 4).

In summary, the NGRNs' need for social support in order to process their feelings during their learning process was seen as vital and this was facilitated by their mentors, both during individual conversations and during group discussions. It was also noted that having time dedicated to these conversations, in an undisturbed setting, is vital as regards supporting the NGRNs during their learning process.

## 5 Discussion

This focused ethnographic study explores the NGRNs' need for professional support and how this need is addressed by their mentors during real‐life work activities. The main theme: *I'm in freefall and in need of practical, social and emotional support when navigating my new role* highlights that NGRNs rely on the active presence and support of their clinical mentors within care units. The findings indicate that clinical mentors can support NGRNs by creating an in situ learning environment and this environment should integrate practical hands‐on support in daily tasks, social and emotional support, all of which are essential for strengthening NGRNs' professional confidence and easing their transition into practice.

The theme, *I am a new nurse and in need of help in comprehending and performing nursing*, shows both the relevance of and demand for NGRNs continuously being practically supported in their day‐to‐day work and engaging, in the moment, in a supportive and critically reflective dialogue together with their clinical mentors whose reasoning is in the best interests of the patient. Interactions between NGRNs and their clinical mentors emphasise a learning process that involves communication, reflection and meaning, being in line with Vygotsky (1978) sociocultural perspective which holds that interacting is key to learning. In the present study, the NGRNs often displayed both insecurity and the need to have their thoughts confirmed, which is in line with Billett et al. (2018), who proposes that the key aspect of supporting newcomers (here the NGRNs) is either an activity or systematic engagement, on the individual level, in day‐to‐day work. This type of Work Integrated Learning (WIL) can thus be seen as facilitating organisations in fostering a supportive learning environment for their NGRNs. Whereby clinical mentors use reflective dialogues as a pedagogical tool, together with the NGRNs, for integrating theory and practice (Berndtsson et al. 2020) so as to gain some insight into whether or not they need to increase their skills in order to perform their day‐to‐day duties. In the present study, NGRNs comprehend and perform nursing in dialogue with their mentors when reasoning around their patients' needs and best interests. The benefit of encouraging a reflective dialogue while the NGRNs are working is further described by Lindblom et al. (2025), and is also seen as vital for professional development that includes planning, prioritising, performing duties, validating clinical judgement and supporting decision‐making. This advocates the need to offer a learning environment wherein clinical mentors are available during every shift.

The present study highlights the fact that the NGRNs needed guidance since they lacked both knowledge of and confidence in procedures, for example, central venous catheter, leaving them both unprepared and unequipped with the skills and knowledge needed to perform independently, which has also been identified in earlier studies (Eklund et al. 2025; Tast et al. 2024). We found that the NGRNs to varying degrees wanted to be guided in new procedures, to be asked questions, or to have their thoughts confirmed by their clinical mentors, and to be assured as regards protecting their patients' safety. They were thus providing care and protecting their patients' safety in accordance with what they had learned during more traditional training and previous educational settings, and they were reliant on expertise in nursing practice which Benner et al. (2009) refer to as the social embeddedness of knowledge. Our study additionally emphasised that the NGRNs lacked the contextual awareness, as beginners, for understanding their actions, as well as practical experience of situations. According to Benner (1982), beginners rely on guidelines in order to gain sufficient knowledge for evaluating patient care situations (i.e., recognition), and they also need some experience of care situations before being able to work independently with only guideline support. Offering the NGRNs the continuous support of their clinical mentors in their day‐to‐day work is thus seen as important in terms of being a method of mentoring ‘in situ’ experience. The finding also shows that the NGRNs often needed support when prioritising tasks. In line with Benner (1982), NGRNs have begun to recognise recurring meaningful patterns, while still requiring assistance in determining what is most important in a given situation. This study also shows how clinical mentors who are experienced nurses helped to reduce the NGRNs' ‘tunnel vision’ arising from a lack of the contextual understanding needed for the functional prioritising of procedures and activities. This suggests that mentorship is an essential function for NGRNs to become integrated into, while functionally contributing to day‐to‐day practice at the care unit.

The theme, *I am undergoing a learning process‐ in need of a trust‐based relationship*, shows that reflective mentorship can play an important part in socially and emotionally supporting the NGRNs' learning process. The relevance of NGRNs processing their feelings of being anxious about their personally‐deficient learning, and about performing well without mistakes, requires having conversations in a safe place and in trust‐based dialogue with their clinical mentors. The creation of mentorship as a ‘safe space’ and an integrative aspect of the process of building professional trust and independence indicates the importance of mentorship during the socialisation process while more importantly being a precondition for learning the more practical and instrumental task of nursing. The mentors conducted both individual and group sessions where photos were used as a pedagogical tool for the NGRNs' reflections on their feelings as newcomers. Benner et al. (2009) confirm that advanced beginners (i.e., NGRNs) can become emotionally‐drained during the initial period of wanting to protect their patients' safety. One review also shows that NGRNs need to have the possibility of sharing their experiences, achievements and frustrations, and to be affirmed in the challenges they experience during their first year: Here, mentorship was found to be the most important support strategy both for retaining NGRNs and for their wellbeing (Melissant et al. 2024). A further study confirms that mentors play a crucial part in NGRNs' needs as regards talking about their feelings in the complex working environment they are entering (Kallerhult Hermansson et al. 2024). This study shows that NGRNs appreciated the possibility of sharing their achievements during mentor conversations, and also that, in order to develop, concrete feedback on their work was required from their clinical mentors. Consequently, to function as clinical mentors, mentors need, at least to some extent, to be present and involved in everyday activities in daily work. Clinical mentorship thus becomes embedded and performed within an established and functional community of practice, however, adding to such practice by safeguarding the allocation of time, space and resources to continues ongoing learning opportunities in situ (Lave and Wenger 1991). This finding emphasises that mentorship is a key aspect of enabling NGRNs to be supported individually in their day‐to‐day work, which means in person‐centric learning, and that it is vital to create resilience.

In sum, NGRNs benefit from the support of their trusted clinical mentors in terms of practical and more hands‐on support as well as social and emotional support, and this is mutually vital. This allows them to navigate, act, and learn the nursing role, which is necessary, and mentorship could legitimise the NGRNs during the process by which the novice moves away from peripheral participation towards the centre of the community (Lave and Wenger 1991). As our study shows, and with previous studies also confirming the benefits of mentorship, this is the key to the NGRNs' adaptation to the hospital setting (Alharbi et al. 2023), with increased confidence and competence (Giltenane et al. 2026). Furthermore, even though our results come from two specific care unit settings, many of the features and characteristics conditioning nursing in this context are to some degree generic and shared by working nurses more widely. This in turn suggests that insights from this study might be used to inform the development of more generic and structured mentorship programmes for NGRNs, whereby clinical mentors' practical experiences, situated professional socialising, and the impact of organisational restraints become an integrated and essential part of the training and mentorship occurring in the day‐to‐day work of NGRNs. Further, knowledge is needed in order to develop a structured mentorship programme for NGRNs, whereby the clinical mentors' perspectives and organisational perspectives are also essential when it comes to successfully implementing this kind of programme, and the need to further study mentorship in relation to retaining NGRNs.

### 5.1 Strengths and Limitations

This is a focused ethnographic study that includes mentoring at two healthcare units. However, the units and situations in focus were carefully selected to represent mentorship within a hospital culture in Sweden, potentially being transferrable to another context with careful consideration. One strength of this study was the researcher's preunderstanding (Andreassen et al. 2020) of how and when mentoring situations occur, while also maintaining her independence, as the researcher was not affiliated with the care units. Although data collection was relatively short, it focused on mentoring situations during day‐to‐day work, generating rich and detailed descriptions. As noted by Atkinson and Pugsley (2005), particularly informative situations can produce extensive and valuable data, with 1 h of observation potentially yielding several hours of written work. Furthermore, only one male participated in this study, and this could be seen as a limitation. However, we interpret this as a normal gender distribution in nursing, with the participants being selected based on the structured mentorship that had been established within the organisation rather than having a structured selection based on gender. The scope of the NGRNs' wide variety of experience as new nurses could be seen as a limitation since there could be some variation in the need for support. Hence, the distribution of experience could also be seen as a strength reflecting the fact that the NGRNs have different levels of experience during their first year, and mentoring must adapt to the individual NGRN's experience.

### 5.2 Implications for Policy and Practice

The findings from this study indicate that structured mentorship can improve the learning environment of NGRNs and that it can constitute a sustainable working environment for them. Decision‐makers and managers can combine this knowledge in designing and implementing effective mentoring programmes, which is of interest in retaining newcomers and also acts as a way of retaining experienced nurses while providing qualitative and safe healthcare.

## 6 Conclusions

This study provides insights into the challenging experiences faced by NGRNs on entering hospital settings, needing practical, social and emotional support from clinical mentors. The findings demonstrate that NGRNs require guidance in their day‐to‐day work, often provided in the moment by clinical mentors through dialogue and hands‐on support aimed at helping them to comprehend and perform their nursing duties. They also need more structured learning opportunities. Furthermore, NGRNs need to establish trust‐based relationships with their mentors, facilitated through feedback and personal recognition, in order to strengthen their learning and sense of belonging at the care unit. Overall, this allows them to navigate, act and learn the nursing role and can be fostered through close social interaction with clinical mentors who possess nursing experience and in‐depth knowledge of the specific care unit.

## Author Contributions

**Pernilla Berndtsson:** conceptualisation, data curation, formal analysis, methodology, validation, visualisation, writing – original draft introduction, methods, result, writing – review and editing. **Malin Berghammer:** conceptualisation, validation, writing – original draft. **Lars Walter:** conceptualisation, methodology, formal analysis, supervision, validation, writing – original draft discussion. **Maria Skyvell Nilsson:** conceptualisation, methodology, formal analysis, supervision, validation, writing – review and editing.

## Funding

The authors have nothing to report.

## Conflicts of Interest

The authors declare no conflicts of interest.

## Data Availability

The data is not publicly available because of privacy and ethical considerations in order to secure the confidentiality of the participants.
